# Hepatocellular Carcinoma in Asia: Physician and Patient Perspectives on Surveillance, Diagnosis, and Treatment

**DOI:** 10.1007/s12029-024-01089-5

**Published:** 2024-07-12

**Authors:** Rosmawati Mohamed, Wendy Wang, Tawesak Tanwandee, Irsan Hasan, Cam Phuong Pham, Young-Suk Lim, Sheng-Nan Lu, Murallitharan Munisamy, Thi Thanh Huong Tran, Evy Ratnawati, Wattana Sukeepaisarnjaroen, Mahir Karababa, Chee-Kiat Tan

**Affiliations:** 1 University of Malaya, Kuala Lumpur, Malaysia; 2 Liver Disease Prevention and Treatment Research Foundation, Taipei, Taiwan; 3 Department of Medicine, Faculty of Medicine Siriraj Hospital, Mahidol University, Bangkok, Thailand; 4 Faculty of Medicine, University Indonesia/Ciptomangunkusumo Hospital, Jakarta, Indonesia; 5 Nuclear Medicine and Oncology Center, Bach Mai Hospital, Department of Nuclear Medicine, HaNoi Medical University, Hanoi, Vietnam; 6 Department of Internal Medicine and Department of Gastroenterology, Asan Medical Centre, University of Ulsan College of Medicine, Seoul, Republic of Korea; 7 Division of Hepatogastroenterology, Department of Internal Medicine, Kaohsiung Chang Gung Memorial Hospital, Kaohsiung, Taiwan; 8 National Cancer Society of Malaysia, Sunway University, 66 Jalan Raja Muda Abdul Aziz, 50300 Kuala lumpur, Malaysia; 9 National Cancer Institute, Hanoi Medical University, Hanoi, Vietnam; 10 Indonesian Cancer Information and Support Center (CISC), Jakarta, Indonesia; 11 Faculty of Medicine and Department of Medicine, Gastrointestinal Unit, Srinagarind Hospital, Khon Kaen University, Khon Kaen, Thailand; 12 Medical Affairs, COR2ED, Basel, Switzerland; 13 Department of Gastroenterology and Hepatology, Singapore General Hospital, Academia, 20 College Road, Singapore, 169856 Singapore

**Keywords:** Hepatocellular Carcinoma, Asia, Diagnosis, Therapeutics, Surveys and questionnaires

## Abstract

**Purpose:**

In several Asian countries, hepatocellular carcinoma (HCC) is a leading cause of cancer deaths. HCC risk factors in Asia differ from those elsewhere and are changing with the treatment landscape as systemic treatment options increase. This study was conducted to gain insight from physicians and patients into HCC screening, diagnosis, and treatment strategies in Indonesia, Korea, Malaysia, Singapore, Taiwan, Thailand, and Vietnam.

**Methods:**

Two cross-sectional, anonymized, online surveys were completed between July and December 2022 by physicians diagnosing and treating HCC (55 questions on risk factors, surveillance, diagnosis, and treatment) and patients ≥ 18 years old diagnosed with HCC (36 questions on disease knowledge, quality of life, and experiences of diagnosis and treatment).

**Results:**

Responses were received from 276 physicians in all 7 countries and 130 patients in Thailand, Taiwan, and Vietnam. From the physician’s perspective, surveillance programs are widespread but identify insufficient HCC cases; only 18% are early-stage HCC at diagnosis. From the patient’s perspective, knowledge of risk factors increases after diagnosis, but few seek support from patient associations; patients would benefit from better communication from their doctors. Treatment affordability and side effects are key issues for patients.

**Conclusions:**

Awareness of the risk factors for HCC should be raised in primary care and the general population, and surveillance should identify early-stage HCC. Because patients rely on their doctors for support, doctors should better understand their patients’ needs, and patients could be supported by trained nurses or case managers. Programs are needed to increase patients’ access to proven HCC treatments.

**Supplementary Information:**

The online version contains supplementary material available at 10.1007/s12029-024-01089-5.

## Introduction

Estimated in 2020 to have caused 830,000 deaths, liver cancer is the third leading cause of death due to cancer worldwide and is the leading cause of death due to cancer in both men and women in Mongolia, Thailand, and Cambodia [1]. In 2020, 72.5% of the world's cases of liver cancer were in Asia [1, 2]. Most (75–85%) cases are hepatocellular carcinoma (HCC) [1], of which the most important causes, in Asia, are chronic infection by hepatitis B virus (HBV) or hepatitis C virus (HCV) [3]. Other risk factors include aflatoxin exposure, excessive alcohol intake, smoking, obesity, type 2 diabetes mellitus, and non-alcohol-related steatohepatitis (NASH). While the average consumption of alcohol continues to grow, excessive alcohol consumption will remain an important risk factor [2], but the epidemiology of HCC in Asia is changing. The prevalence of non-alcohol-related fatty liver disease (NAFLD) is rising [4], and that of HCC caused by metabolic factors, including metabolic syndrome, obesity, and NASH, is increasing rapidly too [2, 5].

These changes may be accompanied by changes in the treatment landscape in Asia as systemic treatment options for HCC increase. In the most recent international guidelines, the new combination of atezolizumab + bevacizumab is now preferred over tyrosine kinase inhibitors for the first-line (1L) treatment of advanced HCC [6, 7], and it already features in several recent Asian treatment guidelines [3, 8, 9]. We wanted to know whether this changing treatment landscape is accompanied by changing treatment practice and, in turn, changes in diagnostic approaches and surveillance strategies in Asia. Therefore, we conducted this survey study to gain insight from physicians and patients into the strategies currently used in Asia to screen for, diagnose, and treat HCC. No similar research has been conducted on the perspectives of both patients with HCC and their treating physicians on an Asian-wide basis.

## Methods

**Fig. 1:**
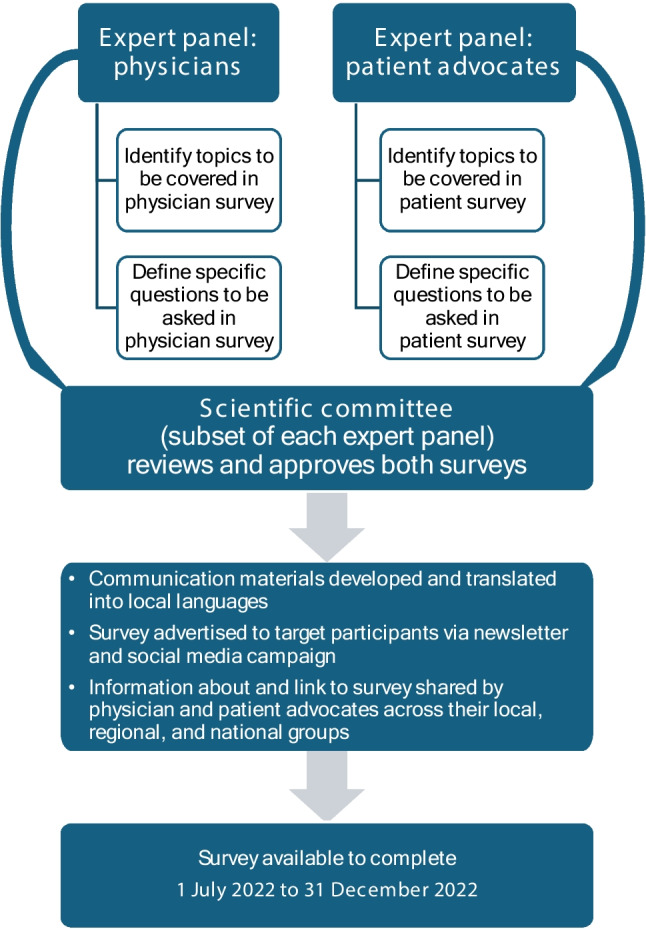
Development, validation, and administration of the survey

We are a group of physicians specializing in HCC (authors RM, TT, IH, PPC, Y-SL, S-NL, C-KT) and members of patient advocate groups whose purpose is to support patients with HCC (authors WW, MM, HTTT, ER, WS) in Indonesia, Korea, Malaysia, Singapore, Taiwan, Thailand, and Vietnam. These 7 countries form the focus of our study, which comprised two cross-sectional, anonymized, online surveys hosted by Qualtrics XM (available from https://www.qualtrics.com/): one of 55 questions for physicians diagnosing and treating HCC, and one of 36 questions for patients diagnosed with HCC and ≥ 18 years old. Additional details of the development, validation, and administration of the survey are provided in Fig. 1.

Most questions were closed; some included options for open responses. The physician survey asked about risk factors for HCC and the surveillance landscape in Asia, HBV and HCV elimination strategies in Asia, HCC diagnosis, treatment of early and intermediate HCC and of advanced HCC, and use of multidisciplinary tumor boards (MTBs). The patient survey asked about patients’ knowledge of the disease, their quality of life (QoL), their diagnosis and treatment journeys, and their access to treatments for HCC. Both surveys asked questions about the respondents’ characteristics. The complete questionnaires are provided in the Online Resource.

The study populations comprised, for the physician survey, peers and professional contacts of the physician authors and, for the patient survey, patients known to the authors’ patient advocacy groups. The surveys were sent to potential respondents by email and accompanied by a flyer that indicated the organizations conducting and supporting the study (COR2ED and Roche, respectively) and stated the aim of the study (i.e., to evaluate the management of HCC in Asia). The sample size was not pre-specified. Because the link to the survey was distributed via multiple avenues (see Fig. 1) we are unable to determine with any certainty how many people received or opened the link and, therefore, we are unable to provide a response rate. The surveys were available to be completed anonymously online between 1 July and 31 December 2022. Because of poor internet access in some locations, some patient surveys were administered on paper and the data subsequently entered online.

## Results

We received 276 responses from physicians in all 7 countries and 130 from patients in Thailand, Taiwan, and Vietnam (we received too few responses for analysis from patients in the other 4 countries). Respondents’ characteristics are listed in Table 1. Most physicians who participated in the survey are hepatologists, except in Vietnam, where medical oncologists predominate. Most physicians work in large hospitals, and many see large numbers of patients. Respondents most commonly make 6–9 HCC diagnoses per month, except in Korea, where 20–49 diagnoses predominate.

**Table 1:** Survey respondents’ characteristics

	**Indonesia**	**Korea**	**Malaysia**	**Singapore**	**Taiwan**	**Thailand**	**Vietnam**	**Total**
**Physician respondents, n**	38	31	43	33	34	65	32	276
Hepatologist or gastroenterologist, %	71	65	58	58	62	97	19	66
Oncologist, %	13	16	19	24	15	0	50	17
Interventional radiologist, %	5	13	9	9	3	2	13	7
Hepatobiliary surgeon, %	11	6	7	9	3	0	16	7
Nurse, %	0	0	0	0	18	0	3	3
Other, %	0	0	7	0	0	2	0	1
Primary institution^a^								
Large national hospital or medical center, %	55	58	58	64	71	49	63	59
Mid-sized or regional hospital, %	42	39	33	33	26	34	31	34
Private clinic, %	3	3	5	3	3	11	3	5
Rural or local hospital, %	0	0	0	0	0	2	3	1
Other, %	0	0	5	0	0	2	0	1
HCC patients seen per month								
1–5, %	8	0	44	6	9	38	25	22
6–9, %	28	14	29	49	23	25	3	24
10–19, %	45	29	16	39	9	17	34	26
20–49, %	16	48	9	6	32	15	22	20
50–99, %	3	3	2	0	21	5	13	6
≥ 100, %	0	6	0	0	6	0	3	2
HCC diagnoses per month								
1–5, %	0	3	16	3	9	6	3	6
6–9, %	47	29	67	76	53	75	41	58
10–19, %	32	19	9	21	21	11	34	20
20–49, %	21	42	7	0	6	6	9	12
50–99, %	0	6	0	0	12	2	6	3
≥ 100, %	0	0	0	0	0	0	6	1
**Patient respondents, n**	–	–	–	–	40	59	31	130
Age								
18–29 years, %	–	–	–	–	3	2	3	2
30–39 years, %	–	–	–	–	3	5	3	4
40–49 years, %	–	–	–	–	28	8	23	18
50–59 years, %	–	–	–	–	25	25	29	26
60–69 years, %	–	–	–	–	10	37	29	27
70–79 years, %	–	–	–	–	30	10	10	16
≥ 79 years, %	–	–	–	–	3	12	3	7
Sex: male, %	–	–	–	–	70	64	74	68
Time since diagnosis								
< 1 month, %	–	–	–	–	3	0	6	2
1–3 months, %	–	–	–	–	8	17	19	15
4–5 months, %	–	–	–	–	5	8	32	13
6–9 months, %	–	–	–	–	3	10	13	8
12–15 months, %	–	–	–	–	5	3	3	4
16–19 months, %	–	–	–	–	0	3	0	3
20–24 months, %	–	–	–	–	3	3	6	4
2–3 years, %	–	–	–	–	23	10	10	14
4–5 years, %	–	–	–	–	0	10	3	5
≥ 5 years, %	–	–	–	–	48	34	6	32
HCC stage^b^ at diagnosis								
Early, %	–	–	–	–	53	63	35	53
Intermediate, %	–	–	–	–	30	17	39	26
Advanced, %	–	–	–	–	5	0	13	5
Don’t know, %	–	–	–	–	8	17	10	12
Other, %	–	–	–	–	5	3	3	4
HCC stage^b^ currently								
Early, %	–	–	–	–	30	39	29	34
Intermediate, %	–	–	–	–	28	15	48	27
Advanced, %	–	–	–	–	13	5	16	10
Don’t know, %	–	–	–	–	13	27	3	17
Other, %	–	–	–	–	18	14	3	12
HCC management satisfaction score, 1–5								
5 (extremely satisfied), %	–	–	–	–	43	75	32	55
4, %	–	–	–	–	35	19	32	27
3, %	–	–	–	–	20	5	35	17
2, %	–	–	–	–	3	0	0	1
1 (extremely unsatisfied), %	–	–	–	–	0	2	0	1
Impact of HCC on daily life								
Significant	–	–	–	–	18	44	58	39
Moderate	–	–	–	–	48	27	29	34
Minimal	–	–	–	–	13	14	0	10
None	–	–	–	–	23	15	13	17

^a^Large national hospital or medical center: average 750 beds, range 600–900 beds; mid-sized or regional hospital: average 300 beds, range: 200–400 beds; private clinic: average 25 beds, range 15–35 beds; rural or local hospital: average 110 beds, range 80–140 beds

^b^Stage as communicated by the physician to the patient

HCC, hepatocellular carcinoma

Most patient participants are male. Half of them are younger than 59 years (patient respondents’ mean age at the time of diagnosis was 56.5 years and at the time of the survey, 58.9 years), have been living with HCC for 2 years or more, and had been diagnosed with early-stage HCC; 61% currently have early- or intermediate-stage HCC (Table 1). Most patients consider the disease to have a “significant” or “moderate” impact on their QoL, and most are “satisfied” or “extremely satisfied” with the management of their HCC to date.

Selected key responses in addition to those described below are provided in the Online Resource.

### Physician Survey: HCC Surveillance

Awareness of a national HCC surveillance program or database (Supplementary Tables HCPS1 and HCPS2) differs by country and appears to be poor in Indonesia, Malaysia, and Vietnam. Surveillance is conducted mainly by hepatologists or gastroenterologists (Supplementary Table HCPS5). Hepatitis B and C, fatty liver (NAFLD) or NASH, and cirrhosis are recognized as risk factors for HCC in each country (Supplementary Table HCPS3), and patients with these risk factors (except NAFLD/NASH) are widely screened (Supplementary Table HCPS4). Surveillance programs targeting patients with NAFLD or NASH are sparse. Very few respondents believe surveillance programs identify ≥ 50% of cases of HCC (Table 2); around half believe they identify ≥ 20% of cases. The main barriers to more widespread surveillance are consistently considered to be cost and funding issues and a lack of patient awareness or healthcare provider knowledge about HCC surveillance. Surveillance is impeded in Indonesia by a lack of access to specialist care, in Taiwan and Thailand by a lack of manpower, and in Vietnam by a lack of well-structured local guidelines.

**Table 2:** Percentage of respondents’ patients with HCC initially identified by a surveillance program and percentages of patients in groups at risk of HCC

**Patients identified, %**	**Country**							
	**Indonesia**	**Korea**	**Malaysia**	**Singapore**	**Taiwan**	**Thailand**	**Vietnam**	**Total**
	**Respondents, %**							
≤ 10	32	0	28	0	21	22	53	22
10– < 20	34	39	16	27	21	34	25	28
20– < 40	29	55	23	33	21	20	9	26
40– < 50	3	6	19	18	18	9	9	12
50– < 70	3	0	9	12	12	6	3	7
70– < 90	0	0	5	9	3	9	0	4
> 90	0	0	0	0	6	0	0	1
**Group at risk**	**Patients, %**							
HBV infected	32	37	36	33	42	35	46	37
Liver cirrhosis	37	18	22	25	16	20	15	22
HCV infected	19	24	17	14	23	12	15	17
Fatty liver or NASH	10	9	16	17	6	12	10	12
Alcohol-related cirrhosis	6	12	8	10	10	20	13	12
Other	0	0	1	0	3	0	0	1

HBV, hepatitis B virus; HCC, hepatocellular carcinoma; HCV, hepatitis C virus; NASH, non-alcohol-related steatohepatitis

**Fig. 2:**
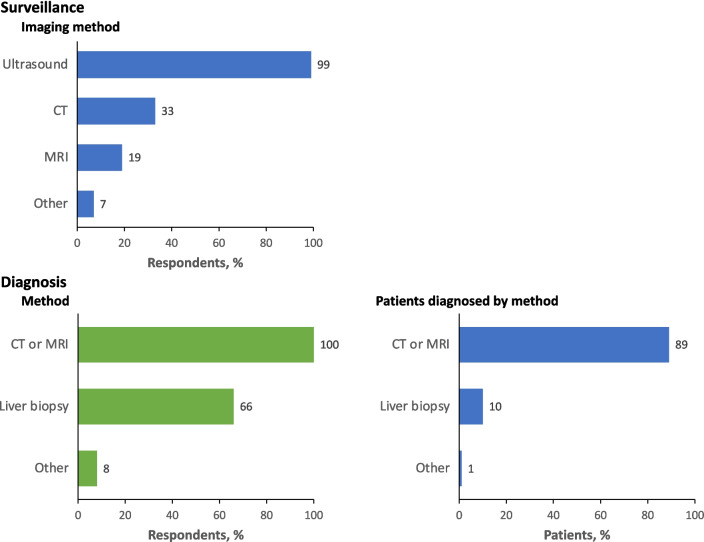
Diagnostic methods used for surveillance and diagnosis of HCC in Asia. CT, computed tomography; MRI, magnetic resonance imaging

The primary imaging method used for surveillance is ultrasound (Fig. 2), which is widely accessible, and its use is mostly reimbursed (Supplementary Tables HCPS6–8). Biomarkers are widely used, and similar percentages of respondents have access to and use specific biomarkers (Supplementary Tables HCPS9–11).

### Physician Survey: HCC Prevention

Respondents consider the population at risk of HCC to be those with HBV infection, liver cirrhosis, HCV infection, NAFLD or NASH, or alcohol-related cirrhosis (Table 2). All the countries have a national vaccination program to prevent HBV infection (Supplementary Table HCPS12), and HCV infection is treated and widely subjected to elimination programs.

### Physician Survey: Diagnosis of HCC

HCC is most commonly diagnosed at intermediate stage (Barcelona Clinic Liver Cancer [BCLC] stage B, in 29%) or advanced stage (BCLC stage C, in 31%). Diagnosis of early HCC (BCLC stage 0 or A) is rare (18%). Almost all diagnosis is by multiphasic computed tomography (CT) or magnetic resonance imaging (MRI), supported by serum total alfa fetoprotein (AFP); the use of liver biopsy is rare (Fig. 2).

Respondents believe that early diagnosis of HCC could be improved (Table 3) by conducting screening for HBV and HCV infection in everyone at risk, linking HCC surveillance to specialist care for those at risk, detecting underlying liver disease earlier, and engaging primary care providers and providing them with the knowledge and tools to identify at-risk patients.

**Table 3:** Physicians’ opinions on factors in need of improvement to facilitate early diagnosis of HCC and patients’ perspectives of factors in need of improvement during the diagnosis period

**Factor to improve**	**Physician respondents, %**							
	**Indonesia**	**Korea**	**Malaysia**	**Singapore**	**Taiwan**	**Thailand**	**Vietnam**	**Total**
HBV or HCV screening in all at risk	97	94	100	73	74	88	81	87
HCC surveillance with referral to specialist care	92	97	91	91	59	88	72	85
Earlier detection of underlying liver disease	95	81	95	85	74	78	66	82
Empower primary care providers to identify those at risk	76	77	100	76	71	91	69	82
Access to MTB	74	52	77	48	29	62	56	58
Other	3	0	7	0	3	3	0	3
	**Patient respondents, %**							
	**Male**			**Female**			**Total**	
Communication by doctor	30			38			33	
Speed of diagnosis	27			40			31	
Access to imaging	15			33			20	
Availability of patient association or group support	18			23			19	
Other^a^	9			8			8	

^a^Opportunity to share experiences in group meetings

HBV, hepatitis B virus; HCC, hepatocellular carcinoma; HCV, hepatitis C virus; MTB, multidisciplinary tumor board

Staging is done primarily by the BCLC system (Supplementary Table HCPS18); the tumor–node–metastasis (TNM) system is also used, to a lesser extent. Besides indicating the extent of HCC and prognosis, staging systems are also used for deciding the best treatment option.

### Physician Survey: Treatment of HCC

For early or intermediate-stage HCC, the primary objective is treatment with curative intent to prolong survival. Also important are preventing recurrence, improving QoL, and preserving liver function. Most treatment options are available, used (Table 4), and reimbursed (as applicable) (Supplementary Tables HCPS19 and HCPS20).

**Table 4:** Treatments given by physician respondents to patients with early- or intermediate-stage HCC or advanced HCC

**Treatment**	**Respondents, %**							
	**Indonesia**	**Korea**	**Malaysia**	**Singapore**	**Taiwan**	**Thailand**	**Vietnam**	**Total**
**Early or intermediate HCC**								
Resection	95	97	98	97	97	97	100	97
Local ablative therapy	76	81	88	82	100	94	81	87
TACE	97	68	86	64	94	92	88	86
Transplantation	53	87	67	97	91	68	75	75
Radioembolization or TARE	53	39	70	64	62	62	69	60
Radiation therapy	37	61	44	61	79	49	44	53
**Advanced HCC**								
First line								
Sorafenib	97	100	88	73	97	77	100	89
Lenvatinib	89	97	91	85	97	49	88	81
Atezolizumab + bevacizumab	76	58	63	82	82	54	72	68
Second line^a^								
Regorafenib	84	100	28	76	88	45	88	68
Pembrolizumab	32	81	51	70	82	45	88	56
Nivolumab	87	65	26	52	85	46	6	51
Ramucirumab	55	77	0	58	74	20	28	40
Cabozantinib	18	58	26	73	56	14	6	33
Nivolumab + ipilimumab	18	23	12	42	62	34	9	29

^a^Other treatments (immunotherapy, tyrosine kinase inhibitor, or other) are used by ≤ 7% of respondents

HCC, hepatocellular carcinoma; TACE, transarterial chemoembolization; TARE, transarterial radioembolization

For advanced HCC, key objectives are to prolong survival, control symptoms, and improve QoL. The 1L treatment options available and widely used (Table 4, Supplementary Table HCPS22) are sorafenib, lenvatinib, and atezolizumab + bevacizumab. As of December 2022, atezolizumab + bevacizumab was approved in all surveyed countries but reimbursed and widely used only in Korea (by 58%) and Singapore (by 82%). Despite the lack of reimbursement the combination is widely used in Taiwan (by 82%), Indonesia (76%), Vietnam (72%), and Malaysia (63%) and less so in Thailand (54%). Greater access and availability would increase the use of atezolizumab + bevacizumab as the new standard of care and, in patients for whom immunotherapy is contraindicated, of lenvatinib (Supplementary Table HCPS23). Reasons for switching to systemic therapy are progression to advanced HCC (BCLC stage C) or failure of other treatments.

The most widely available and most used (Table 4) second-line (2L) treatment options for advanced HCC are regorafenib, pembrolizumab, and nivolumab. With greater availability, other systemic treatment options would be more widely used (Supplementary Table HCPS25).

### Physician Survey: Use of MTBs

MTBs (see Supplementary Tables HCPS26–28) are widely used in Asia, as in the rest of the world. They are accessible by 86% of respondents, most commonly in large national hospitals. Most MTBs comprise hepatologists or gastroenterologists, hepatobiliary surgeons, oncologists, interventional radiologists, and radiation oncologists.

### Patient Survey: Disease Knowledge

Since receiving their diagnosis, patients substantially improved their knowledge of the risk factors for HCC (Table 5), with many gaining better understanding and having greater awareness of the roles of key risk factors (Supplementary Table PS1). Other risk factors are overwork or stress and aflatoxin in food. Few (6–12%) patients had no change in their knowledge of the risk factors for HCC after diagnosis. Most patients understand the symptoms of HCC, but few are aware of easy bruising or bleeding and tea-colored urine as symptoms (Supplementary Table PS1).

**Table 5:** Change since diagnosis in patients’ knowledge of risk factors for HCC

**Risk factor**	**Change in knowledge since diagnosis of HCC, % respondents**				
	**Significantly improved**	**Somewhat** **improved**	**Slightly** **improved**	**No change**	**Not aware of** **this factor**
Cirrhosis	50	24	12	11	4
Alcohol consumption	49	25	14	8	4
Hepatitis B or C	45	27	15	7	5
HCV infection	45	27	12	7	9
HBV infection	42	28	12	7	11
Family history	42	26	12	12	8
NASH	40	20	18	7	15
Drug abuse	34	25	19	7	15
Metabolic syndrome^a^	34	26	15	12	12
Blood transfusion, hemodialysis, shared needles	33	25	15	6	21

^a^Obesity, diabetes, arterial hypertension

HBV, hepatitis B virus; HCC, hepatocellular carcinoma; HCV, hepatitis C virus; NASH, non-alcohol-related steatohepatitis

### Patient Survey: The Diagnosis Journey

For most patients, a diagnosis of HCC is confirmed within 3 months of first suspicion (Supplementary Table PS3) in a public hospital (Supplementary Table PS7). Suspicion is raised mainly by a hepatologist or gastroenterologist (Supplementary Table PS6). Most patients find communication about their diagnosis “excellent” or “good” and believe they received the correct tests, with clear explanations about those tests (Supplementary Tables PS4, PS5, and PS9). Rarely did patients seek support from a patient association or support group (Supplementary Table PS8). One third of patients would have liked better communication to help them understand the disease and also a more expedient diagnosis (Table 3).

### Patient Survey: The Treatment Journey

Most patients can name the first treatment they received and had already received 1 or 2 treatments when they completed the survey (Supplementary Tables PS10 and PS11). In general, patients are happy with communication from their doctor about treatment choice (Supplementary Table PS12). During treatment, most patients rely on their doctor for support (Supplementary Table PS17). The most important outcomes of treatment are cure and prolonging life (Supplementary Table PS16).

For the 16% of patients who stopped a treatment, the decision to stop was made mainly by the patient and because the treatment is not affordable or has unwanted side effects (Supplementary Tables PS13–15). Side effects are experienced by 56% of patients, and before starting a treatment 68% of patients were aware of possible side effects (Supplementary Tables PS18 and PS19). The side effects most challenging to patients are fatigue, nausea or vomiting, loss of appetite, physical weakness, and stomachache. Patients believe that treatment of HCC could be improved not only by better management of their side effects but also by greater access to treatments (Supplementary Table PS20). Nevertheless, they are optimistic: 76% believe they “have access to the best suitable treatment” (Supplementary Table PS23). In terms of financial support, 61% are covered by health insurance and 53% find treatments unaffordable (Supplementary Tables PS21 and PS22).

## Discussion

Our study has provided important insights into current strategies for the surveillance, diagnosis, and treatment of HCC used in 7 Asian countries. This is the first time that such research has been conducted on the perspectives of both patients with HCC and treating physicians in Asia. Our physician survey findings indicate that HCC is diagnosed at early stage in fewer than 20% of patients. It is, therefore, evident that surveillance strategies and their effectiveness need to be improved. It is not clear whether differences between countries in the effectiveness of surveillance are due to different surveillance strategies or levels of awareness of the existence of a surveillance program, but examination of the strategies used in the relatively more successful countries in our study (e.g., Singapore and Taiwan, where 21% of respondents believe surveillance to identify 50% or more of cases) may provide clues to potential improvements in the other countries. In all 7 countries, there appears to be a need to increase the role of general practitioners or primary care providers in raising suspicion of HCC, perhaps initially by increasing their awareness of screening those at risk of HCC and the need for HCC surveillance programs. A need for improved education of physicians and care coordination has also been suggested by a survey in Pakistan, which showed gaps in physicians’ knowledge and inconsistent practices in managing chronic hepatitis B [10]. Monitoring at-risk populations, clinician education, and national guidelines have already been highlighted as regional priorities for liver cancer control in Asia [11], with political and public awareness and lifestyle risk management crucial for HCC control in the Asia–Pacific region [12].

Our study supports the suggestion that awareness about HCC risk factors should also be raised among the general population, given that knowledge about risk factors improved in 88–94% of patients after HCC diagnosis. More people need to know that they should undergo screening for chronic liver disease to identify those at risk of HCC. A recent large-scale Asian study also showed a need for better patient and public education about liver diseases in general [13]. Older surveys of Chinese and Southeast Asian Canadians [14] and Vietnamese immigrants in the USA [15] indicated a need for improved education about HBV, and a survey of Asian Americans with chronic hepatitis C showed that early antiviral treatment and routine HCC surveillance are needed in that population [16]. For patients, the importance of early detection of HCC is illustrated by our finding that the disease has a “significant” or “moderate” impact on QoL for 39% and 34% of patients, respectively, despite most respondents (61%) having only early- or intermediate-stage disease at the time of the survey. The importance of early detection was also highlighted in 2011 by a survey of physicians’ views of emerging technologies in HCC [17].

Our patient survey shows that although the time from first suspicion to diagnosis is 3 months or less for most patients, it is 4–12 months for 19% and more than 12 months for 12% of patients. Indeed, 31% of patients would prefer a more expedient diagnosis. Internationally, diagnostic imaging practices in HCC differ substantially by region [18], and for European and Asian patients with HCC there are geographic differences in demographics, etiology, diagnostic methods, and treatment approaches [19, 20]. Diagnostic and treatment strategies may therefore need to be region-specific [20]. We believe that for patients in Asia, the diagnosis journey could be streamlined by promoting use of ultrasound imaging, which is readily available, inexpensive, and noninvasive. Its use, with serum biomarkers, is supported by Asian guidelines [3, 8, 9, 21, 22], and in the parts of Asia surveyed, ultrasound and serum biomarkers are widely used for surveillance (Fig. 2 and Online Resource, Table HCPS10).

Remarkably, few patients seek support from a patient association or support group during the diagnosis period. It may be that such support is lacking or that patients are reluctant to seek it, even if available, because of the stigma associated with a diagnosis of HCC. Such stigma may deter patients from joining a support group. We believe that this barrier can be removed by providing case managers or social care workers to support patients.

The biggest change in the treatment landscape in HCC has been for advanced disease. Although sorafenib as a 1L treatment is readily accessible, other treatments such as immunotherapy are only partly reimbursed, and patients need help with treatment costs. Such help could be provided via the MTB by including a social care worker in the team, who can request and coordinate treatment funding support on patients’ behalf. Help could also be obtained through market access programs from the pharmaceutical companies or health insurance companies. At the time of the survey, atezolizumab + bevacizumab was widely used, notably in Taiwan despite a lack of reimbursement there. Our physician survey shows that greater access and availability would increase the use of atezolizumab + bevacizumab as the standard of care and of lenvatinib for the population for whom immunotherapy is contraindicated, a finding which suggests that treatment choice is strongly influenced by the out-of-pocket cost to the patient.

Our patient survey shows that patients have a high level of satisfaction with the management of their HCC, perhaps partly attributable to the less negative impact of the newer treatments on patients’ QoL. Nevertheless, management of side effects needs to be improved. Another area for improvement is in the provision of information and advice by medical staff to facilitate shared decision making. To achieve this aim, patients could be supported by counselling by a trained and certified member of the nursing staff, or case manager.

Comparison of the latest guidelines from the American Association for the Study of Liver Diseases (AASLD) [23], European Association for the Study of the Liver (EASL) [24], and Asian Pacific Association for the Study of the Liver (APASL) [25] reveals a global convergence in HCC treatment strategies across the EU, US, and Asia. However, variations exist because of regional differences in healthcare infrastructure and disease prevalence. In the EU, emphasis is placed on a multidisciplinary approach, whereas the US prioritizes a combination of surgical and systemic therapies, and in Asia traditional practices are integrated into modern treatment approaches. Despite the convergence in guidelines, access to innovative therapies remains challenging, particularly in Asia, where new treatments may become available only some time after their approval in the US and EU.

Our study has several limitations. Because of the way the survey was distributed, we are unable to determine the exact response rate: survey links were distributed through multiple channels, therefore it is not clear how many potential respondents received or opened the survey invitations. The questionnaires may not comprehensively cover all aspects of HCC management. Although they included questions about risk factors, surveillance, diagnosis, treatment, and quality of life, other relevant areas may not have been addressed. Future studies should include a broader range of questions to capture a more complete picture of HCC management practices and patient experiences. Administration of the patient survey faced challenges due to variable internet access across the surveyed countries. In some regions, limited access to the internet led to the use of printed surveys that were later digitized. This process might have introduced inconsistencies or data entry errors. Despite these efforts, we received very few responses in some countries and therefore had insufficient data for a comprehensive analysis of patient perspectives in those regions. The sample size, especially for the patient survey, was relatively small and may not be representative of the broader HCC patient population in Asia. Indeed, the patient survey may have been completed mainly by relatively younger, more engaged, and fitter patients who are more internet savvy and therefore do not fully represent the populations targeted. In addition, the findings we report here are specific to the 7 countries in which the survey was administered and may not be generalizable to Asia as a whole. Therefore, it will be useful to have further research with larger and more representative samples to validate and expand upon our findings.

## Conclusions

Awareness should be raised in both primary care and the general population in Asia about the risk factors for HCC, and surveillance should be improved to enable diagnosis of HCC at an early stage and thus increase the chances of curative treatment.

Patients rely on their doctors for support, and doctors should better understand their patients’ concerns and needs (e.g., managing side effects) and help patients better understand physicians’ roles and perspectives. Patient education and support could be improved by including trained nurses or case managers in the multidisciplinary care team.

Greater effort is needed to create programs to increase patients’ access to HCC treatment options shown to prolong survival.

### Supplementary Information

Below is the link to the electronic supplementary material.

Supplementary file1 (DOCX 117 KB)

## Data Availability

No datasets were generated or analysed during the current study.
