# Supplementary material for: Hepatocellular Carcinoma in Asia: Physician and Patient Perspectives on Surveillance, Diagnosis, and Treatment
Source: J Gastrointest Cancer. 2024 Jul 12;55(3):1333–44. doi: 10.1007/s12029-024-01089-5 (PMC11347480; doi:10.1007/s12029-024-01089-5)
Supplement: Supplementary file 1 — Supplementary file1 (DOCX 117 KB) [file 12029_2024_1089_MOESM1_ESM.docx]

**Supporting Information**

**Journal of Gastrointestinal Cancer**

**Hepatocellular carcinoma in Asia: physician and patient perspectives on surveillance, diagnosis, and treatment**

Rosmawati Mohamed, Wendy Wang, Tawesak Tanwandee, Irsan Hasan, Cam Phuong Pham, Young-Suk Lim, Sheng-Nan Lu, Murallitharan Munisamy, Tran Thi Thanh Huong, Evy Ratnawati, Wattana Sukeepaisarnjaroen, Mahir Karababa, Chee-Kiat Tan

**Correspondence**

Chee-Kiat Tan, Department of Gastroenterology and Hepatology, Singapore General Hospital, Academia, Level 3, 20 College Road, Singapore 169856.

email: [tan.chee.kiat@singhealth.com.sg](mailto:tan.chee.kiat@singhealth.com.sg).

**1 The Questionnaires**

**1.1 Health Care Practitioner (HCP) Survey**

**LANGUAGES:** English, Korean, Thai, Bahasa Melayu, Bahasa, Vietnamese, Traditional Chinese

**Dear Participant:**

Please complete this short survey assessing the diagnosis and treatment of **hepatocellular carcinoma (HCC)** in select countries across the Asia Pacific region.

We expect the questionnaire will take 20-30 minutes to complete. Your individual responses are confidential and will not be shared with other participants; responses will be aggregated and used to develop recommendations to improve HCC management in the Asia Pacific region. We look forward to receiving your feedback.

***Section 1: Your role and HCC workload***

**Q1.1 In which country do you practice?**

- Indonesia
- Korea
- Malaysia
- Singapore
- Taiwan
- Thailand
- Vietnam

**Q1.2 What is your primary specialty?**

- Hepatologist / Gastroenterologist
- Oncologist
- Interventional Radiologist
- Hepatobiliary Surgeon
- Nurse
- Other (specify): _____________

**Q1.3 How would you describe your primary institution?**

- Large national hospital or medical centre
- Mid-sized or regional hospital
- Rural or local hospital
- Private practice clinic
- Other (specify): _____________

**Q1.4 Approximately how many patients with HCC do you personally see per month?**

- None (0)
- 1–5 HCC patients per month
- 6–9 HCC patients per month
- 10–19 HCC patients per month
- 20–49 HCC patients per month
- 50–99 HCC patients per month
- 100+ HCC patients per month

**Q1.5 Approximately how many patients do you personally diagnose with HCC per month?**

- None (0)
- 1–5 patients diagnosed with HCC per month
- 6–9 patients diagnosed with HCC per month
- 10–19 patients diagnosed with HCC per month
- 20–49 patients diagnosed with HCC per month
- 50–99 patients diagnosed with HCC per month
- 100+ patients diagnosed with HCC per month

***Section 2: Monitoring and surveillance for early identification of patients with HCC***

**Q2.1 Is there a national surveillance programme for HCC in your country?**

- Yes
- Yes, but limited to specialists
- No
- I do not know

**Q2.2 Is there a national surveillance database for HCC in your country?**

- Yes
- No
- I do not know

**Q2.3 Which of these populations do you consider to be at risk for HCC in your country?** (*Select all that apply*)

- Hepatitis C (HCV)
- Fatty liver induced / nonalcoholic steatohepatitis (NASH)
- Alcohol-related cirrhosis
- Hepatitis B (HBV)
- Liver cirrhosis
- Other (specify): ________

**Q2.4 In your country, is there a surveillance programme for the at risk population(s) that you selected?** (*Select a response for each population*)

|  | **Yes** | **No** |
| --- | --- | --- |
| Hepatitis C (HCV) | o | o |
| Fatty liver induced / nonalcoholic steatohepatitis (NASH) | o | o |
| Alcohol-related cirrhosis | o | o |
| Hepatitis B (HBV) | o | o |
| Liver cirrhosis | o | o |

**The next group of questions will assess how surveillance for HCC is organised in your country.**

**Q2.5 Who is conducting surveillance for HCC in your country?** (*Select all that apply*)

- Primary Care Providers / General Practitioners
- Hepatologist / Gastroenterologists
- Other(s) (please specify): ________

**Q2.6 As a general practice, which types of medical imaging is/are used to screen for HCC surveillance?** (*Select all that apply*)

- - Ultrasound
  - Computed Tomography (CT)
  - Magnetic Resonance (MR)
  - Other(s) (please specify): ________
  - None

**Q2.7 Which of these types of medical imaging do you have access to for HCC surveillance screening ?** (*Select all that apply*)

- Ultrasound
- Computed Tomography (CT)
- Magnetic Resonance (MR)
- Other(s) (please specify): ________
- None

**Q2.8 Is this type of medical imaging reimbursed in your country when used to screen for HCC?** (*Select a response for each type of imaging*)

|  | **Yes** | **No** | **Not sure** |
| --- | --- | --- | --- |
| Ultrasound | o | o | o |
| Computed tomography (CT) | o | o | o |
| Magnetic resonance (MR) | o | o | o |
| Other | o | o | o |

**Q2.9 As a general practice, which biomarker(s)/tumour marker(s) is/are used for HCC surveillance?** (*Select all that apply*)

- - AFP-L3
  - AFP
  - DCP/PIVKA-II
  - Other(s) (specify): ___________
  - None

**Q2.10 How often are biomarker(s)/tumour marker(s) used as a screening modality for HCC surveillance in your country?**

- Always
- Often
- Sometimes
- Rarely
- Never

**Q2.11 Which of these biomarker(s)/tumour marker(s) do you have access to?** (*Select all that apply*)

- - AFP-L3
  - AFP
  - DCP/PIVKA-II
  - Other(s) (specify): ___________
  - None

**Q2.12 Is/are the biomarker(s)/tumour marker(s) below reimbursed in your country?**

|  | **Yes** | **No** | **Not sure** |
| --- | --- | --- | --- |
| AFP-L3 | o | o | o |
| AFP | o | o | o |
| DCP/PIVKA-II | o | o | o |
| Other | o | o | o |

**Q2.13 Approximately what proportion of your patients diagnosed with HCC are initially identified by a surveillance programme?**

- <5%
- 5–<10%
- 10–<20%
- 20–<40%
- 40–<50%
- 50–<70%
- 70–<90%
- >90%

**Q2.14 What are the 3 main barriers preventing more widespread surveillance for HCC in your country?** (*1 = most important reason, 2 = second most important reason, 3 = third most important reason*)

| 1 |  |
| --- | --- |
| 2 |  |
| 3 |  |

**Q2.15 In your opinion, what is needed to improve surveillance for HCC in your country?**

___________

***Section 3: Diagnosis of HCC***

**Q3.1 Considering the population at risk for HCC in your country, which percentage belong to the following groups?** (*The total must equal 100%*)

|  | **% of at risk HCC patient population** |
| --- | --- |
| Hepatitis C (HCV) | __ % |
| Fatty liver induced / nonalcoholic steatohepatitis (NASH) | __ % |
| Alcohol-related cirrhosis | __ % |
| Hepatitis B (HBV) | __ % |
| Liver cirrhosis | __ % |
| Other | __ % |
| Total | % |

**Q3.2 Is there a national programme for HBV (hepatitis B) or HCV (hepatitis C) vaccination in your country?**

- Yes – for neonatal HBV vaccination
- Yes – for catch-up HBV vaccination
- Yes – for neonatal HCV vaccination
- Yes – for catch-up HCV vaccination
- Yes – other (specify): ________
- No
- Not sure

**Q3.2a Is there a national programme for each of the following in your country?**

|  | **Select** | **Optional comments** |
| --- | --- | --- |
| National programme for **HCV eradication**? | Yes / No / Not sure |  |
| National programme for **HBV eradication**? | Yes / No / Not sure |  |
| National programme for **alcohol abstinence?** | Yes / No / Not sure |  |
| Other national prevention programmes? | Yes / No / Not sure |  |

**Q3.3 Is the HCC biopsy and pathology analysis reimbursed in your country?**

- Yes
- No
- Not sure

**Q3.4 Thinking about the HCC patients you see, what percentage of these patients are diagnosed at each stage of disease?** (*The total must equal 100%*)

__ % Early HCC (BCLC-0/A)

__ % Intermediate HCC (BCLC-B)

__ % Advanced HCC (BCLC-C)

__ % Late-stage HCC (BCLC-D)

**Q3.5 Which methods are used to diagnose a patient with HCC in your country?** (*Select all that apply*)

- Multiphasic CT or MR imaging
- Liver biopsy
- Other (please specify): ________

**Q3.5a What percent of HCC patients in your country would you estimate are diagnosed using each of the following approaches?** (*The total may be more than 100% if more than one method is used in a specific patient*)

___ % of HCC patients diagnosed using multiphasic CT or MR imaging

___ % of HCC patients diagnosed using liver biopsy

___ % of HCC patients diagnosed using other (from Q3.5)

**Q3.6 Which of the following criteria are used to diagnose a patient with HCC in your country?** (*Select all that apply*)

- Dynamic imaging (multiphasic CT or MRI scan, contrast enhanced ultrasound)
- Serum total AFP level
- Serum AFP-L3 level
- Serum PIVKA-II/DCP level
- Histology
- Other (please specify): ________

**Q3.6a How would you rank order these criteria in terms of their importance in diagnosing a patient with HCC in your country?** (*1 = Most important, 2 = Second most important, etc.*)

___________

**Q3.7 Considering the diagnosis of HCC in your country:**

**Q3.7a Who is typically the *first* healthcare provider raising the suspicion of HCC?**

- General practitioner/primary healthcare provider
- Hepatologist/Gastroenterologist
- Oncologist
- Radiologist
- Hepatobiliary Surgeon
- Other (specify) _______

**Q3.7b Where is the diagnosis for HCC most commonly conducted?**

- Large national hospital
- Mid-sized hospital
- Rural hospital
- Private practice clinic
- Other (specify): ________

**Q3.7c Are there diagnosis guidelines for HCC in your country?**

- Yes (please specify which one): ________
- No

**Q3.8 What do you believe needs to be improved regarding EARLY diagnosis of HCC in your country?** (*Select all that apply*)

- Earlier detection of the underlying liver disease
- HBV/HCV screening in everyone at risk (e.g for Hepatitis B those born prior to the implementation of HBV immunization)
- Engage primary care providers to empower them with the knowledge and tools to identify those at risk of HCC
- Better HCC surveillance programme and provide linkage to care for those at risk of HCC
- Improved access to Multi-Disciplinary Tumour (MDT) Boards
- Other (specify): ___________

**Q3.8a How would you rank order the following needs in terms of their importance in terms of improvements needed for EARLY diagnosis of HCC in your country?** (*1 = Most important, 2 = Second most important, etc.*)

___________

***Section 4: Disease staging and treatments***

**Q4.1 Which staging system(s) for HCC is (are) used in your country?** (*Select all that apply*)

- BCLC
- TNM
- CLIP
- JIS
- Milan
- Other (specify): ___________
- None

**Q4.2 In addition to staging the disease and assessment of response to treatment, are you using these staging systems for any other purposes?**

___________

**Q4.3 What are the 3 main objectives of HCC treatment?**

**Q4.3a For early and intermediate stage HCC** (*Provide the most important objective in the first row and any other important objectives in the second and third rows*)

1. ____________
2. ____________
3. ____________

**Q4.3b For Advanced stage of HCC?** (*Provide the most important objective in the first row and any other important objectives in the second and third rows*)

1. ____________
2. ____________
3. ____________

**Q4.4a Which of the following treatment options are available for early and intermediate stage HCC patients in your country?** (*Select all that apply*)

| Resection |
| --- |
| Transplantation |
| Local ablative therapy |
| TACE |
| Radioembolisation / TARE |
| Radiation therapy |
| Other (specify): ________ |

**Q4.4b Is each of the following a treatment given to your early and intermediate stage HCC patients?** (*Select a response in each row*)

| **Treatment selected in Q4.4a** | **Given to your  early and intermediate stage HCC patients?** | **Reimbursed for  early and intermediate stage HCC patients?** |
| --- | --- | --- |
|  | Yes / No | Yes / No / Not sure |

**Q4.4c If this treatment became available in your country, would you *like to use it* for the treatment of early and intermediate stage HCC patients?** (*Select a response in each row*)

| **Treatment not selected in Q4.4a** | **Yes** | **No** | **Not sure** |
| --- | --- | --- | --- |
|  | o | o | o |

**Q4.5a Which of the following treatment options are available as a 1^st^ line treatment option for advanced stage HCC patients in your country?** (*Select all that apply*)

| sorafenib |
| --- |
| lenvatinib |
| atezolizumab + bevacizumab |
| Other immunotherapy (specify): ________ |
| Other TKI (specify): ________ |
| Other (specify): ________ |

**Q4.5b Is each of the following a 1^st^ line treatment given to your advanced stage HCC patients?** (*Select a response in each row*)

| **Treatment selected in Q4.5a** | **Given as  1^st^ line treatment to your advanced stage HCC patients?** | **Reimbursed for 1^st^ line treatment of advanced stage HCC patients?** |
| --- | --- | --- |
|  | Yes / No | Yes / No / Not sure |

**Q4.5c If this treatment became available in your country, would you *like to use it* for the 1^st^ line treatment of advanced stage HCC patients?** (*Select a response in each row*)

| **Treatment not selected in Q4.5a** | **Yes** | **No** | **Not sure** |
| --- | --- | --- | --- |
|  | o | o | o |

**Q4.6a Which of the following treatment options are available as a 2^nd^ line treatment option for advanced stage HCC patients in your country?** (*Select all that apply*)

| regorafenib |
| --- |
| ramucirumab |
| cabozantinib |
| pembrolizumab |
| nivolumab |
| nivolumab + ipilimumab |
| Other immunotherapy (specify): ________ |
| Other TKI (specify): ________ |
| Other (specify): ________ |

**Q4.6b Is each of the following a 2^nd^ line treatment given to your advanced stage HCC patients?** (*Select a response in each row*)

| **Treatment selected in Q4.6a** | **Given as  2^nd^ line treatment to your  advanced stage HCC patients?** | **Reimbursed for 2^nd^ line treatment of advanced stage HCC patients?** |
| --- | --- | --- |
|  | Yes / No | Yes / No / Not sure |

**Q4.6c If this treatment became available in your country, would you *like to use it* for the 2^nd^ line treatment of advanced stage HCC patients?** (*Select a response in each row*)

| **Treatment not selected in Q4.6a** | **Yes** | **No** | **Not sure** |
| --- | --- | --- | --- |
|  | o | o | o |

**Q4.7 What are the top 3 key criteria to switch to systemic therapy for HCC?** (*Rank 1: more important, 3: less important*)

1. ____________
2. ____________
3. ____________

**The next section of questions will focus on multi-disciplinary tumour (MDT) boards in your country.**

**Q4.8 Do you have access to an MDT at your centre or in other centres in your country?**

- Yes
- No

**Q4.9 If “Yes”, please select the option below that best describes your access to an MDT**

- MDT is established at my centre/hospital
- MDT is done via virtual discussion of suspected or diagnosed cases before referral to a tertiary centre
- All suspected or diagnosed HCC patients are referred to a tertiary centre where there is an MDT
- Other (specify): ___________

**Q4.10 Which specialties participate in the MDT board?** (*Select all that apply*)

- - Oncologist
  - Hepatologist/Gastroenterologist
  - Hepatobiliary Surgeons
  - Interventional Radiologist
  - Nuclear medicine physician
  - Radiation oncologist
  - Pathologist
  - Immunologist
  - Other (specify): _______

**Q4.11 Is there a national treatment guideline for HCC in your country?**

- Yes
- No

**Q4.12 Which international treatment guidelines for HCC is the reference in your country?** (*Select all relevant ones*)

- ESMO
- ASCO
- NCCN
- AASLD
- EASL
- Other (specify): _________
- Not sure
- None

**Q4.13 What do you feel could be improved regarding the treatment of advanced HCC in your country?** (*Select all that apply*)

- Access to oncologist in MDT
- Access to systemic treatment at your centre/hospital
- Budget impact analysis to show the cost effectiveness of systemic treatments in advanced HCC
- National guidelines on patients who may benefit from systemic treatments
- Improved access to MDTs
- Others (specify): ___________

**1.2 Patient Survey**

**LANGUAGES:** English, Korean, Thai, Bahasa Melayu, Bahasa, Vietnamese, Traditional Chinese

**Dear Participant:**

Please complete this short survey assessing the diagnosis and treatment of hepatocellular carcinoma (HCC) in a number of countries across the Asia Pacific region.

We expect the questionnaire will take 20-30 minutes to complete. Your individual responses are confidential and will not be shared with other participants; responses will be aggregated and used to develop recommendations for the improvements to HCC management in the Asia Pacific region. We look forward to receiving your feedback.

***Section 1: Tell us about yourself***

**Q1.1 In which country do you live?**

- Indonesia
- Korea
- Malaysia
- Singapore
- Taiwan
- Thailand
- Vietnam

**Q1.2 What is your age?**

- <18 years
- 18–29 years
- 30–39 years
- 40–49 years
- 50–59 years
- 60–69 years
- 70–79 years
- >79 years

**Q1.3** **What is your gender?**

- Female
- Male
- Prefer not to say

**Q1.4 How long has it been since you were first diagnosed with HCC?**

- Less than 1 month
- 1 to 3 months
- 4 to 5 months
- 6 to 9 months
- 12 to 15 months
- 16 to 19 months
- 20 to 24 months
- 2 to 3 years
- 4 to 5 years
- 5 years or longer

**Q1.5 At which stage of the disease were you when first diagnosed with HCC**

- Early-stage HCC
- Intermediate stage HCC
- Advanced stage HCC
- I do not know
- Other (specify):

**Q1.6 What is the stage of your disease currently?**

- Early-stage HCC
- Intermediate stage HCC
- Advanced stage HCC
- I do not know
- Other (specify):

**Q1.7 What is your overall level of satisfaction with the management of your HCC since diagnosis?**

| **Extremely satisfied**  **5** | **4** | **3** | **2** | **Extremely**  **unsatisfied**  **1** |
| --- | --- | --- | --- | --- |
| o | o | o | o | o |

**Q1.8 Why? Please explain your above rating.**

___________

**Q1.9 Since you were diagnosed with HCC, how has your knowledge of the following risk factors *changed* (if at all)?** (*Select a response in each row*)

|  | **Significantly improved since HCC diagnosis** | **Somewhat improved since HCC diagnosis** | **Slightly improved since HCC diagnosis** | **No change since HCC diagnosis** | **Not aware of role this plays in HCC** |
| --- | --- | --- | --- | --- | --- |
| Your knowledge of the **role of Hepatitis** **B/C** in HCC? | o | o | o | o | o |
| Your knowledge of the **role of drug abuse** in HCC? | o | o | o | o | o |
| Your knowledge of the **role alcohol consumption** in HCC? | o | o | o | o | o |
| Your knowledge of the **role of metabolic syndrome** (obesity, diabetes, arterial hypertension) in HCC? | o | o | o | o | o |

**Q1.10 In addition to the above, what other risk factors are you aware of for HCC?**

___________

**Q1.11 Based on your understanding, are each of the following symptoms of HCC?** (*Select a response in each row*)

|  | **Yes** | **No** | **Not sure** |
| --- | --- | --- | --- |
| Is **chronic liver disease** a symptom of HCC? | o | o | o |
| Is **tea-coloured urine** a symptom of HCC? | o | o | o |
| Is **loss of appetite** a symptom of HCC? | o | o | o |
| Is **unintended weight loss** a symptom of HCC? | o | o | o |
| Is **fatigue** a symptom of HCC? | o | o | o |
| Is **jaundice** a symptom of HCC? | o | o | o |
| Is **swelling in the abdomen and legs** a symptom of HCC? | o | o | o |
| Is **easy bruising or bleeding** a symptom of HCC? | o | o | o |
| Is **abdominal pain** a symptom of HCC? | o | o | o |

**Q1.12 In addition to the above, what other symptoms of HCC are you aware of?**

___________

**Q1.13 Since you were diagnosed with HCC, how has your awareness of the following risk factors *changed* (if at all)?** (*Select a response in each row*)

|  | **Significantly improved since HCC diagnosis** | **Somewhat improved since HCC diagnosis** | **Slightly improved since HCC diagnosis** | **No change since HCC diagnosis** | **Not aware of role this plays in HCC** |
| --- | --- | --- | --- | --- | --- |
| Your awareness of **family history** as a risk factor for HCC? | o | o | o | o | o |
| Your awareness of **HCV (hepatitis C virus) infection** as a risk factor for HCC? | o | o | o | o | o |
| Your awareness of **HBV (hepatitis B virus) infection** as a risk factor for HCC? | o | o | o | o | o |
| Your awareness of **NASH (Non-alcoholic steatohepatitis)** as a risk factor for HCC | o | o | o | o | o |
| Your awareness of **Cirrhosis** as a risk factor for HCC | o | o | o | o | o |
| Your awareness of **Blood transfusion / Hemodialysis / shared needles** as a risk factor for HCC | o | o | o | o | o |

**Q1.14** In addition to the above, are there any **other risk factors** that you know play a role in your HCC? If yes, please explain.

___________

**Q1.15 What is the impact of the HCC disease in your daily life today?**

- Significant
- Moderate
- Minimal
- None

**Q1.16 Please describe the impact of HCC on your daily life.**

___________

***Section 2: This section will focus on your experience during the diagnosis of your HCC***

**Q2.1 How long did it take from first suspicion until confirmation of an HCC diagnosis?**

- <1 month
- 1–3 months
- 4–6 months
- 7–12 months
- >12 months

**Q2.2 How did you find the communication with your doctor / practice during the diagnosis period?**

- Excellent
- Good
- Neither good nor bad
- Poor
- There was no communication

**Q2.3 Do you feel that you received the correct medical/laboratory tests during your consultations that led to your HCC diagnosis?**

- Yes
- No
- I do not know

**Q2.4 Please elaborate.**

___________

**Q2.5 Can you please tell us more about your journey that led to your HCC diagnosis?**

**Q2.5a Who first mentioned the suspicion of HCC?**

- Family members or friends
- My general doctor
- A radiologist
- A surgeon
- An oncologist
- A hepatologist/gastroenterologist
- Other (specify): __________

**Q2.5b Where did your HCC diagnosis occur?**

- Public hospital
- Private Hospital
- Other (specify): __________

**Q2.5c Did you contact any patient associations or groups to help you during the diagnosis?**

- Yes (specify which ones): __________
- No
- I do not know

**Q2.5d Did you feel you received clear information to reach your diagnosis HCC?**

- Yes
- No
- I do not know

**Q2.5e Please elaborate on your responses above.**

___________

**Q2.6 From your perspective, what are the most important areas of improvement for the treatment of HCC? (*Select all that apply*)**

- More streamlined (faster) process of diagnosing HCC
- Greater involvement and support from patient associations / groups
- Improved access to imaging / facility to diagnose the disease
- Improved communications with doctors to help you better understand your disease
- Other (please specify): __________

**Q2.7 Please elaborate on your selection(s) above.**

___________

***Section 3: Tell us more about your treatments for HCC***

**Q3.1 Do you know which treatment you received for your HCC initially?**

- Yes (please specify): __________
- No
- I do not know

**Q3.2 How many different treatments for HCC have you received (excluding surgical treatment)?**

- None (0)
- 1 treatment
- 2 treatments
- 3 treatments
- 4 or more treatments

**Q3.3 How did you find the communication with your doctor / practice regarding the selection of treatment for your HCC?**

- Excellent
- Good
- Neutral
- Poor
- No communication

**Q3.4 Have any of your treatments for HCC been stopped?**

- Yes
- No

**Q3.5 If “Yes”, what was the reason for stopping?** (*Select all that apply*)

- Lack of efficacy
- Side effects
- No treatment available
- New treatment available
- Treatment not affordable
- Availability of clinical trials
- Other (specify): _________

**Q3.6 Who stopped the treatment?**

- You
- Your doctor
- Other (specify): _________

**Q3.7 What is the most important outcome for you when you start a treatment for your HCC?**

- To cure HCC
- To extend your life expectancy
- To have a better quality of life
- To avoid difficult side effects
- Other (specify): _________

**Q3.8 Where do you get support during the treatment of your HCC?** (*Select all that apply*)

- Doctor
- Nurse
- Patient association/support group
- Family member/carer
- Other (specify): _________

**Q3.9 Do you have health insurance coverage?**

- Yes (specify which ones): _________
- No

**Q3.10 If “Yes”, what level or type of health insurance do you have?**

___________

**Q3.11 Can you afford all your HCC treatments?**

- Yes
- No

**Q3.12 Please elaborate.**

___________

**Q3.13 Do you think you have access to the best suitable treatment for your HCC?**

- Yes
- No
- I do not know

**Q3.14 If “No”, what is the reason you believe you do not have access to the most suitable treatment for your HCC?**

___________

**Q3.15 Did you experience side effects with the treatment/s you have taken for your HCC?**

- Yes
- No

**Q3.16 Were you aware of the side effects prior to starting the treatment?**

- Yes
- No

**Q3.17 Which side effects did you find most challenging/problematic?**

___________

**Q3.18 From your perspective, what are the most important areas of improvement for the treatment of HCC?** (*Select all that apply*)

- Improved access to the most suitable treatment
- Improved management of side effects
- Greater involvement and support from patient associations / groups
- Improved access to imaging / facility to monitor the disease
- Improved communications with doctors to help you better understand your disease
- Other (please specify): ___________

**Q3.19 Please elaborate on your selection(s) above.**

___________

**2 Key Survey Findings**

**2.1 Healthcare practitioner survey (HCPS)**

**2.1.1 Monitoring and surveillance for early identification of patients with HCC**

**Table HCPS1** Existence of national HCC surveillance programme in respondent’s country

| **HCC surveillance programme** | **Respondents, %** | | | | | | | |
| --- | --- | --- | --- | --- | --- | --- | --- | --- |
|  | **Indonesia** | **Korea** | **Malaysia** | **Singapore** | **Taiwan** | **Thailand** | **Vietnam** | **Total** |
| Yes | 13 | 100 | 21 | 70 | 74 | 55 | 19 | 49 |
| Yes, but limited to specialists | 71 | 0 | 37 | 0 | 3 | 26 | 25 | 25 |
| No | 16 | 0 | 35 | 24 | 6 | 9 | 41 | 18 |
| Don’t know | 0 | 0 | 7 | 6 | 18 | 9 | 16 | 8 |

**Table HCPS2** Existence of national HCC surveillance database in respondent’s country

| **HCC surveillance database** | **Respondents, %** | | | | | | | |
| --- | --- | --- | --- | --- | --- | --- | --- | --- |
|  | **Indonesia** | **Korea** | **Malaysia** | **Singapore** | **Taiwan** | **Thailand** | **Vietnam** | **Total** |
| Yes | 0 | 100 | 40 | 18 | 79 | 34 | 13 | 39 |
| No | 87 | 0 | 44 | 55 | 3 | 25 | 59 | 38 |
| Don’t know | 13 | 0 | 16 | 27 | 18 | 42 | 28 | 23 |

**Table HCPS3**  Populations considered to be at risk of HCC in respondent’s country

| **Population at risk** | **Respondents, %** | | | | | | | |
| --- | --- | --- | --- | --- | --- | --- | --- | --- |
|  | **Indonesia** | **Korea** | **Malaysia** | **Singapore** | **Taiwan** | **Thailand** | **Vietnam** | **Total** |
| HCV infected | 97 | 100 | 86 | 82 | 97 | 88 | 94 | 91 |
| Fatty liver or NASH | 63 | 52 | 84 | 82 | 71 | 83 | 59 | 72 |
| Alcohol-related cirrhosis | 37 | 65 | 58 | 67 | 82 | 94 | 63 | 69 |
| HBV infected | 100 | 100 | 100 | 100 | 100 | 100 | 100 | 100 |
| Liver cirrhosis | 92 | 81 | 88 | 88 | 91 | 94 | 84 | 89 |
| Other | 0 | 0 | 5 | 9 | 3 | 0 | 0 | 2 |

**Table HCPS4**  Existence of surveillance programme for at-risk population

| **Surveillance programme for population at risk** | **Respondents who said “Yes”, %** | | | | | | | |
| --- | --- | --- | --- | --- | --- | --- | --- | --- |
|  | **Indonesia** | **Korea** | **Malaysia** | **Singapore** | **Taiwan** | **Thailand** | **Vietnam** | **Total** |
| HBV infected | 92 | 100 | 77 | 94 | 97 | 95 | 88 | 92 |
| HCV infected | 87 | 100 | 65 | 55 | 97 | 80 | 56 | 77 |
| Liver cirrhosis | 53 | 81 | 67 | 64 | 68 | 86 | 31 | 67 |
| Alcohol-related cirrhosis | 16 | 65 | 33 | 45 | 35 | 83 | 9 | 45 |
| Fatty liver or NASH | 8 | 3 | 42 | 12 | 21 | 48 | 9 | 24 |
| Other | 0 | 0 | 2 | 6 | 0 | 0 | 0 | 1 |

**2.1.2 Who conducts surveillance for HCC in respondent’s country, and how**

**Table HCPS5**  Agent conducting HCC surveillance in respondent’s country

| **Agent conducting HCC surveillance** | **Respondents, %** | | | | | | | |
| --- | --- | --- | --- | --- | --- | --- | --- | --- |
|  | **Indonesia** | **Korea** | **Malaysia** | **Singapore** | **Taiwan** | **Thailand** | **Vietnam** | **Total** |
| Hepatologist or gastroenterologist | 97 | 100 | 95 | 100 | 100 | 95 | 75 | 95 |
| Primary care provider or GP | 3 | 29 | 28 | 55 | 29 | 52 | 47 | 36 |
| Other | 3 | 0 | 2 | 0 | 3 | 2 | 31 | 5 |

**Table HCPS6**  Medical imaging modality used for HCC screening as part of surveillance

| **Imaging modality** | **Respondents, %** | | | | | | | |
| --- | --- | --- | --- | --- | --- | --- | --- | --- |
|  | **Indonesia** | **Korea** | **Malaysia** | **Singapore** | **Taiwan** | **Thailand** | **Vietnam** | **Total** |
| Ultrasound | 100 | 100 | 100 | 100 | 91 | 100 | 100 | 99 |
| Computed tomography | 29 | 65 | 30 | 36 | 53 | 9 | 38 | 33 |
| Magnetic resonance | 11 | 3 | 19 | 27 | 44 | 8 | 31 | 19 |
| Other | 0 | 0 | 12 | 0 | 9 | 15 | 3 | 7 |

**Table HCPS7**  Medical imaging modality accessible for HCC screening as part of surveillance

| **Imaging modality** | **Respondents, %** | | | | | | | |
| --- | --- | --- | --- | --- | --- | --- | --- | --- |
|  | **Indonesia** | **Korea** | **Malaysia** | **Singapore** | **Taiwan** | **Thailand** | **Vietnam** | **Total** |
| Ultrasound | 100 | 100 | 100 | 100 | 91 | 97 | 100 | 98 |
| Computed tomography | 71 | 74 | 91 | 67 | 88 | 49 | 47 | 68 |
| Magnetic resonance | 50 | 58 | 81 | 73 | 86 | 45 | 41 | 61 |
| Other | 0 | 3 | 2 | 6 | 3 | 0 | 3 | 2 |
| None | 0 | 0 | 0 | 0 | 3 | 3 | 0 | 1 |

**Table HCPS8**  Use of medical imaging modality reimbursed when used for HCC screening

| **Use of imaging modality reimbursed** | **Respondents who said “Yes”, %** | | | | | | | |
| --- | --- | --- | --- | --- | --- | --- | --- | --- |
|  | **Indonesia** | **Korea** | **Malaysia** | **Singapore** | **Taiwan** | **Thailand** | **Vietnam** | **Total** |
| Ultrasound | 100 | 100 | 58 | 27 | 91 | 82 | 50 | 74 |
| Computed tomography | 26 | 3 | 42 | 18 | 62 | 37 | 9 | 30 |
| Magnetic resonance | 24 | 0 | 35 | 18 | 53 | 32 | 3 | 25 |
| Other | 0 | 0 | 0 | 6 | 0 | 0 | 0 | 1 |

Biomarkers are widely used (by 99% overall, **Table HCPS10**), with alfa fetoprotein (AFP) the most common (used by 99%) (**Table HCPS9**). Biomarkers used to a lesser extent are the protein induced by vitamin K deficiency or antagonism II (PIVKA-II, also called des-gamma-carboxy prothrombin [DCP]), used by 33%, and the lens culinaris agglutinin-reactive fraction of AFP (AFP-L3), used by 17% of respondents (**Table HCPS9**). PIVKA-II is accessible to only 9% of respondents in each of Malaysia and Singapore and 15% of those in Thailand; AFP-L3 is accessible to 6% of respondents in Taiwan, 9% in Thailand, 12% in Malaysia, 16% in each of Indonesia and Korea, and 56% in Vietnam (**Table HCPS11**).

**Table HCPS9**  Biomarkers or tumour markers used for HCC surveillance

| **Marker used for surveillance** | **Respondents, %** | | | | | | | |
| --- | --- | --- | --- | --- | --- | --- | --- | --- |
|  | **Indonesia** | **Korea** | **Malaysia** | **Singapore** | **Taiwan** | **Thailand** | **Vietnam** | **Total** |
| AFP | 100 | 100 | 98 | 97 | 100 | 97 | 100 | 99 |
| PIVKA-II (DCP) | 53 | 45 | 9 | 18 | 76 | 14 | 41 | 33 |
| AFP-L3 | 0 | 35 | 14 | 30 | 6 | 9 | 41 | 17 |
| None | 0 | 0 | 0 | 0 | 0 | 3 | 0 | 1 |
| Other | 0 | 0 | 0 | 0 | 0 | 0 | 3 | 0 |

**Table HCPS10**  Frequency of use of biomarkers or tumour markers to screen for HCC as part of surveillance

| **Frequency of use of markers** | **Respondents, %** | | | | | | | |
| --- | --- | --- | --- | --- | --- | --- | --- | --- |
|  | **Indonesia** | **Korea** | **Malaysia** | **Singapore** | **Taiwan** | **Thailand** | **Vietnam** | **Total** |
| Always | 45 | 48 | 35 | 42 | 50 | 32 | 13 | 37 |
| Often | 53 | 52 | 58 | 42 | 44 | 57 | 72 | 54 |
| Sometimes | 0 | 0 | 7 | 15 | 6 | 6 | 16 | 7 |
| Rarely | 3 | 0 | 0 | 0 | 0 | 3 | 0 | 1 |
| Never | 0 | 0 | 0 | 0 | 0 | 2 | 0 | 0 |

**Table HCPS11**  Biomarkers or tumour markers accessible for use by HCPs

| **Marker used for surveillance** | **Respondents, %** | | | | | | | |
| --- | --- | --- | --- | --- | --- | --- | --- | --- |
|  | **Indonesia** | **Korea** | **Malaysia** | **Singapore** | **Taiwan** | **Thailand** | **Vietnam** | **Total** |
| AFP | 100 | 100 | 98 | 94 | 100 | 98 | 100 | 99 |
| PIVKA-II (DCP) | 53 | 45 | 9 | 9 | 79 | 15 | 47 | 34 |
| AFP-L3 | 16 | 16 | 12 | 33 | 6 | 9 | 56 | 19 |
| None | 0 | 0 | 0 | 0 | 0 | 2 | 0 | 0 |

**Table HCPS12** Existence of a national programme for prevention of HCC in the respondent’s country

| **Prevention programme** | **Respondents, %** | | | | | | | |
| --- | --- | --- | --- | --- | --- | --- | --- | --- |
|  | **Indonesia** | **Korea** | **Malaysia** | **Singapore** | **Taiwan** | **Thailand** | **Vietnam** | **Total** |
| HBV eradication | 100 | 100 | 67 | 64 | 76 | 69 | 97 | 80 |
| HCV eradication | 100 | 97 | 72 | 58 | 94 | 78 | 47 | 78 |
| Alcohol abstinence | 0 | 3 | 33 | 21 | 12 | 51 | 16 | 23 |
| Other | 18 | 6 | 16 | 6 | 3 | 15 | 19 | 13 |

**2.1.3 Diagnosis of HCC**

**Table HCPS13** Methods used for diagnosis of HCC in respondent’s country

| **Method used** | **Respondents, %** | | | | | | | |
| --- | --- | --- | --- | --- | --- | --- | --- | --- |
|  | **Indonesia** | **Korea** | **Malaysia** | **Singapore** | **Taiwan** | **Thailand** | **Vietnam** | **Total** |
| Multiphasic CT or MR imaging^a^ | 100 | 100 | 100 | 100 | 100 | 100 | 100 | 100 |
| Liver biopsy^b^ | 76 | 71 | 30 | 67 | 88 | 58 | 88 | 66 |
| Other | 5 | 0 | 5 | 12 | 0 | 2 | 6 | 4 |

^a^ Used in 90% of patients. ^b^ Used in 10% of patients.

**Table HCPS14**  Criteria used for diagnosis of HCC in respondent’s country

| **Criterion used** | **Respondents, %** | | | | | | | |
| --- | --- | --- | --- | --- | --- | --- | --- | --- |
|  | **Indonesia** | **Korea** | **Malaysia** | **Singapore** | **Taiwan** | **Thailand** | **Vietnam** | **Total** |
| Dynamic imaging^a^ | 97 | 100 | 98 | 100 | 97 | 98 | 100 | 99 |
| Serum total AFP | 100 | 100 | 84 | 61 | 82 | 69 | 100 | 83 |
| Histology | 68 | 48 | 51 | 45 | 85 | 78 | 81 | 67 |
| Serum PIVKA-II (DCP) | 63 | 52 | 9 | 0 | 50 | 11 | 53 | 31 |
| Serum AFP-L3 | 3 | 68 | 14 | 6 | 6 | 9 | 56 | 20 |
| Other | 0 | 0 | 0 | 3 | 3 | 2 | 0 | 1 |

^a^ Multiphasic CT or MRI, contrast-enhanced ultrasound.

**Table HCPS15**  HCP who typically first raises suspicion of HCC

| **HCP type** | **Respondents, %** | | | | | | | |
| --- | --- | --- | --- | --- | --- | --- | --- | --- |
|  | **Indonesia** | **Korea** | **Malaysia** | **Singapore** | **Taiwan** | **Thailand** | **Vietnam** | **Total** |
| GP or primary healthcare provider | 8 | 6 | 9 | 9 | 9 | 31 | 22 | 15 |
| Hepatologist or gastroenterologist | 89 | 94 | 86 | 88 | 88 | 65 | 44 | 78 |
| Oncologist | 0 | 0 | 0 | 0 | 0 | 0 | 34 | 4 |
| Radiologist | 0 | 0 | 0 | 3 | 3 | 2 | 0 | 1 |
| Hepatobilliary surgeon | 0 | 0 | 5 | 0 | 0 | 2 | 0 | 1 |
| Other | 0 | 0 | 0 | 0 | 0 | 2 | 0 | 1 |

**Table HCPS16**  The most common setting of diagnosis of HCC

| **Setting** | **Respondents, %** | | | | | | | |
| --- | --- | --- | --- | --- | --- | --- | --- | --- |
|  | **Indonesia** | **Korea** | **Malaysia** | **Singapore** | **Taiwan** | **Thailand** | **Vietnam** | **Total** |
| Large national hospital | 71 | 74 | 70 | 67 | 76 | 43 | 78 | 66 |
| Mid-size hospital | 26 | 26 | 30 | 33 | 18 | 57 | 22 | 33 |
| Rural hospital | 3 | 0 | 0 | 0 | 0 | 0 | 0 | 0 |
| Private practice | 0 | 0 | 0 | 0 | 0 | 0 | 0 | 0 |
| Other | 0 | 0 | 0 | 0 | 6 | 0 | 0 | 1 |

**Table HCPS17**  Existence of guidelines for diagnosis of HCC in respondent’s country

| **Diagnosis guidelines exist** | **Respondents, %** | | | | | | | |
| --- | --- | --- | --- | --- | --- | --- | --- | --- |
|  | **Indonesia** | **Korea** | **Malaysia** | **Singapore** | **Taiwan** | **Thailand** | **Vietnam** | **Total** |
| Yes | 100 | 100 | 47 | 70 | 68 | 94 | 100 | 83 |
| No | 0 | 0 | 53 | 30 | 32 | 6 | 0 | 17 |

**2.1.4 Disease staging and treatments**

**Table HCPS18** HCC staging system used in respondent’s country

| **Staging system** | **Respondents, %** | | | | | | | |
| --- | --- | --- | --- | --- | --- | --- | --- | --- |
|  | **Indonesia** | **Korea** | **Malaysia** | **Singapore** | **Taiwan** | **Thailand** | **Vietnam** | **Total** |
| BCLC | 97 | 100 | 100 | 94 | 94 | 97 | 100 | 97 |
| TNM | 3 | 42 | 16 | 24 | 68 | 12 | 44 | 27 |
| CLIP | 26 | 0 | 0 | 3 | 35 | 11 | 0 | 11 |
| JIS | 0 | 3 | 0 | 0 | 0 | 0 | 6 | 1 |
| Milan | 45 | 0 | 35 | 70 | 24 | 45 | 16 | 36 |
| Other | 0 | 0? | 5 | 0 | 0 | 2 | 0 | 0 |
| None | 5? | 23? | 0 | 0 | 0 | 0 | 0 | 4? |

**Table HCPS19** Treatments available for early and intermediate stage HCC in respondents’ countries

| **Treatment** | **Respondents, %** | | | | | | | |
| --- | --- | --- | --- | --- | --- | --- | --- | --- |
|  | **Indonesia** | **Korea** | **Malaysia** | **Singapore** | **Taiwan** | **Thailand** | **Vietnam** | **Total** |
| Resection | 92 | 97 | 100 | 97 | 97 | 97 | 100 | 97 |
| Local ablative therapy | 76 | 81 | 93 | 82 | 100 | 94 | 81 | 88 |
| TACE | 95 | 68 | 93 | 64 | 94 | 94 | 88 | 87 |
| Transplantation | 50 | 94 | 74 | 97 | 94 | 78 | 78 | 80 |
| Radioembolisation or TARE | 50 | 45 | 79 | 64 | 76 | 71 | 72 | 66 |
| Radiation therapy | 47 | 68 | 70 | 67 | 85 | 62 | 44 | 63 |
| Other | 0 | 0 | 0 | 3 | 0 | 0 | 0 | 0 |

**Table HCPS20**  Treatments for early and intermediate stage HCC that are reimbursed

| **Treatment** | **Respondents, %** | | | | | | | |
| --- | --- | --- | --- | --- | --- | --- | --- | --- |
|  | **Indonesia** | **Korea** | **Malaysia** | **Singapore** | **Taiwan** | **Thailand** | **Vietnam** | **Total** |
| Resection | 63 | 97 | 63 | 33 | 91 | 89 | 94 | 76 |
| TACE | 76 | 65 | 53 | 33 | 91 | 86 | 84 | 71 |
| Local ablative therapy | 11 | 77 | 58 | 33 | 97 | 77 | 78 | 62 |
| Transplantation | 5 | 77 | 37 | 30 | 82 | 37 | 31 | 41 |
| Radiation therapy | 8 | 58 | 28 | 27 | 76 | 45 | 44 | 40 |
| Radioembolisation or TARE | 5 | 32 | 40 | 30 | 18 | 31 | 47 | 29 |
| Other | 0 | 0 | 0 | 0 | 0 | 0 | 0 | 0 |

In Indonesia, which had the lowest number of responses (53% vs 67–97% in the other countries) for the use of liver transplantation, greater availability of donor livers would increase transplantation rates (**Table HCPS21**).

**Table HCPS21**  Treatments for early and intermediate stage HCC that would be used if available

| **Treatment** | **Respondents, %** | | | | | | | |
| --- | --- | --- | --- | --- | --- | --- | --- | --- |
|  | **Indonesia** | **Korea** | **Malaysia** | **Singapore** | **Taiwan** | **Thailand** | **Vietnam** | **Total** |
| Resection | 26 | 3 | 0 | 3 | 3 | 3 | 0 | 5 |
| Transplantation | 53 | 6 | 19 | 3 | 0 | 15 | 22 | 17 |
| Local ablative therapy | 42 | 16 | 5 | 18 | 0 | 6 | 16 | 14 |
| TACE | 21 | 23 | 7 | 36 | 6 | 6 | 6 | 14 |
| Radioembolisation or TARE | 45 | 45 | 21 | 27 | 15 | 20 | 19 | 26 |
| Radiation therapy | 37 | 32 | 26 | 24 | 9 | 17 | 44 | 26 |

**Table HCPS22**  First-line treatments available for advanced HCC

| **Treatment** | **Respondents, %** | | | | | | | |
| --- | --- | --- | --- | --- | --- | --- | --- | --- |
|  | **Indonesia** | **Korea** | **Malaysia** | **Singapore** | **Taiwan** | **Thailand** | **Vietnam** | **Total** |
| Sorafenib | 97 | 100 | 95 | 88 | 100 | 83 | 100 | 93 |
| Lenvatinib | 89 | 97 | 93 | 94 | 97 | 54 | 91 | 84 |
| Atezolizumab + bevacizumab | 82 | 65 | 74 | 85 | 85 | 63 | 78 | 75 |
| Other immunotherapy | 3 | 0 | 0 | 12 | 32 | 6 | 3 | 8 |
| Other TKI | 8 | 0 | 5 | 6 | 3 | 3 | 6 | 4 |
| Other treatment | 0 | 0 | 14 | 3 | 0 | 6 | 6 | 5 |

**Table HCPS23** First-line treatments for advanced HCC that would be used if available

| **Treatment** | **Respondents, %** | | | | | | | |
| --- | --- | --- | --- | --- | --- | --- | --- | --- |
|  | **Indonesia** | **Korea** | **Malaysia** | **Singapore** | **Taiwan** | **Thailand** | **Vietnam** | **Total** |
| Sorafenib | 88 | 0 | 50 | 25 | 0 | 36 | 0 | 52 |
| Lenvatinib | 100 | 100 | 33 | 100 | 100 | 70 | 100 | 78 |
| Atezolizumab + bevacizumab | 100 | 91 | 64 | 100 | 80 | 71 | 100 | 83 |

**Table HCPS24**  Second-line treatments available for advanced HCC

| **Treatment** | **Respondents, %** | | | | | | | |
| --- | --- | --- | --- | --- | --- | --- | --- | --- |
|  | **Indonesia** | **Korea** | **Malaysia** | **Singapore** | **Taiwan** | **Thailand** | **Vietnam** | **Total** |
| Regorafenib | 76 | 100 | 42 | 79 | 88 | 52 | 91 | 71 |
| Pembrolizumab | 18 | 81 | 72 | 70 | 82 | 34 | 84 | 59 |
| Nivolumab | 74 | 65 | 37 | 55 | 88 | 51 | 6 | 53 |
| Ramucirumab | 47 | 81 | 7 | 64 | 76 | 26 | 31 | 43 |
| Cabozantinib | 3 | 61 | 35 | 76 | 71 | 22 | 6 | 36 |
| Nivolumab + ipilimumab | 8 | 29 | 21 | 42 | 65 | 40 | 9 | 31 |
| Other immunotherapy | 8 | 0 | 2 | 6 | 6 | 3 | 0 | 4 |
| Other TKI | 3 | 0 | 5 | 6 | 0 | 2 | 3 | 3 |
| Other treatment | 0 | 0 | 9 | 6 | 0 | 9 | 0 | 4 |

**Table HCPS25**  Second-line treatments for advanced HCC that would be used if available

| **Treatment** | **Respondents, %** | | | | | | | |
| --- | --- | --- | --- | --- | --- | --- | --- | --- |
|  | **Indonesia** | **Korea** | **Malaysia** | **Singapore** | **Taiwan** | **Thailand** | **Vietnam** | **Total** |
| Nivolumab | 45 | 82 | 67 | 87 | 75 | 69 | 90 | 75 |
| Regorafenib | 100 | 0 | 64 | 57 | 50 | 55 | 100 | 67 |
| Nivolumab + ipilimumab | 53 | 73 | 68 | 63 | 75 | 56 | 90 | 66 |
| Cabozantinib | 76 | 75 | 61 | 75 | 60 | 45 | 83 | 65 |
| Pembrolizumab | 65 | 100 | 67 | 90 | 50 | 47 | 100 | 63 |
| Ramucirumab | 71 | 83 | 48 | 42 | 88 | 56 | 91 | 62 |

**Table HCPS26**  Access to an MTB

| **MTB is available** | **Respondents, %** | | | | | | | |
| --- | --- | --- | --- | --- | --- | --- | --- | --- |
|  | **Indonesia** | **Korea** | **Malaysia** | **Singapore** | **Taiwan** | **Thailand** | **Vietnam** | **Total** |
| Yes | 61 | 97 | 84 | 100 | 97 | 86 | 84 | 86 |
| No | 39 | 3 | 16 | 0 | 3 | 14 | 16 | 14 |

**Table HCPS27**  MTB discussions: respondent’s centre or hospital has an established MTB, cases are discussed in virtual meetings before suspected or diagnosed cases are referred to a tertiary centre, or suspected or diagnosed cases are referred to a tertiary centre with an MTB

| **Type of case discussion** | **Respondents, %** | | | | | | | |
| --- | --- | --- | --- | --- | --- | --- | --- | --- |
|  | **Indonesia** | **Korea** | **Malaysia** | **Singapore** | **Taiwan** | **Thailand** | **Vietnam** | **Total** |
| By established MTB at own institution | 46 | 60 | 81 | 70 | 85 | 52 | 81 | 67 |
| By virtual meeting | 21 | 10 | 6 | 3 | 12 | 11 | 15 | 11 |
| By MTB at another institution | 18 | 30 | 6 | 27 | 3 | 34 | 4 | 19 |
| Other | 14 | 0 | 8 | 0 | 0 | 4 | 0 | 4 |

**Table HCPS28**  Composition of MTB by specialty

| **Specialty** | **Respondents, %** | | | | | | | |
| --- | --- | --- | --- | --- | --- | --- | --- | --- |
|  | **Indonesia** | **Korea** | **Malaysia** | **Singapore** | **Taiwan** | **Thailand** | **Vietnam** | **Total** |
| Hepatology or Gastroenterology | 74 | 97 | 79 | 100 | 94 | 80 | 72 | 84 |
| Hepatobiliary surgery | 61 | 94 | 81 | 100 | 94 | 77 | 84 | 83 |
| Oncology | 58 | 97 | 81 | 94 | 85 | 80 | 84 | 82 |
| Interventional radiology | 61 | 81 | 77 | 79 | 94 | 78 | 78 | 78 |
| Radiation oncology | 55 | 65 | 42 | 70 | 97 | 49 | 59 | 60 |
| Pathology | 37 | 29 | 28 | 52 | 76 | 28 | 56 | 41 |
| Nuclear medicine | 11 | 32 | 9 | 42 | 53 | 25 | 47 | 29 |
| Immunology | 8 | 0 | 5 | 3 | 9 | 5 | 2 | 7 |
| Other | 0 | 0 | 2 | 0 | 6 | 3 | 0 | 2 |

The most commonly used international treatment guidelines are those of the American Association for the Study of Liver Diseases (AASLD^1–3^) (75% of respondents), the European Association for the Study of the Liver (EASL^4^) (71%), and the US National Comprehensive Cancer Network (NCCN^5^) (55%) (**Table HCPS29**). Respondents in each country indicated that treatment of advanced HCC should be improved, potentially by knowing the cost-effectiveness of systemic treatments (85% of respondents), having access to systemic treatments (72%), availability of national guidelines that identify the patients who may benefit from systemic treatment (70%), having an oncologist on the MTB (58%), and improving access to MTBs (51%) (**Table HCPS30**).

**Table HCPS29** International HCC treatment guideline used in respondent’s country

| **International guideline used** | **Respondents, %** | | | | | | | |
| --- | --- | --- | --- | --- | --- | --- | --- | --- |
|  | **Indonesia** | **Korea** | **Malaysia** | **Singapore** | **Taiwan** | **Thailand** | **Vietnam** | **Total** |
| AASLD | 92 | 84 | 42 | 76 | 88 | 83 | 63 | 75 |
| EASL | 79 | 77 | 67 | 42 | 76 | 83 | 63 | 71 |
| NCCN | 45 | 81 | 47 | 55 | 71 | 26 | 97 | 55 |
| ESMO | 63 | 0 | 37 | 45 | 68 | 14 | 59 | 38 |
| ASCO | 3 | 0 | 40 | 24 | 65 | 28 | 66 | 32 |
| Other | 0 | 10 | 12 | 6 | 15 | 8 | 9 | 8 |
| Not sure or none | 5 | 0 | 2 | 6 | 0 | 8 | 0 | 4 |

**Table HCPS30**  HCPs’ opinions on factors needing improvement in the treatment of advanced HCC in their country

| **Factor to improve** | **Respondents, %** | | | | | | | |
| --- | --- | --- | --- | --- | --- | --- | --- | --- |
|  | **Indonesia** | **Korea** | **Malaysia** | **Singapore** | **Taiwan** | **Thailand** | **Vietnam** | **Total** |
| Knowledge of cost effectiveness of systemic treatments | 95 | 97 | 84 | 91 | 82 | 78 | 72 | 85 |
| Access to systemic treatment at own institution | 92 | 71 | 79 | 61 | 44 | 83 | 63 | 72 |
| National guideline specifying who should receive systemic treatment | 87 | 87 | 74 | 61 | 44 | 78 | 47 | 70 |
| Access to oncologist in MTB | 55 | 48 | 74 | 39 | 47 | 58 | 75 | 58 |
| Access to MTB | 58 | 35 | 77 | 42 | 24 | 51 | 59 | 51 |
| Other | 3 | 0 | 2 | 0 | 12 | 2 | 0 | 3 |

**References**

1. AASLD guidelines. Available from https://www.aasld.org/practice-guidelines/management-hepatocellular-carcinoma. Accessed March 2023.

2. Heimbach JK, Kulik LM, Finn RS, Sirlin CB, Abecassis MM, Roberts LR, Zhu AX, Murad MH, Marrero JA (2018) AASLD guidelines for the treatment of hepatocellular carcinoma. Hepatology 67:358-380. https://doi.org/10.1002/hep.29086.

3. Marrero JA, Kulik LM, Sirlin CB, Zhu AX, Finn RS, Abecassis MM, Roberts LR, Heimbach JK (2018) Diagnosis, staging, and management of hepatocellular carcinoma: 2018 practice guidance by the American Association for the Study of Liver Diseases. Hepatology 68:723-750. https://doi.org/10.1002/hep.29913.

4. European Association for the Study of the Liver (EASL) (2018) EASL clinical practice guidelines: management of hepatocellular carcinoma. J Hepatol 69:182-236. https://doi.org/10.1016/j.jhep.2018.03.019. Erratum (2019) in: J Hepatol 70:817.

5. Benson III AB, et al. NCCN guidelines version 1.2023: hepatocellular carcinoma. Available from https://www.nccn.org/professionals/physician_gls/pdf/hcc.pdf. Accessed March 2023.

**2.2 Patient survey (PS)**

**2.2.1 Patients’ characteristics**

**Table PS1** Patients’ understanding of the symptoms of HCC

| **Symptom** | **Awareness, % respondents** | | |
| --- | --- | --- | --- |
|  | **Yes** | **No** | **Not sure** |
| Chronic liver disease | 74 | 10 | 16 |
| Unintended weight loss | 68 | 15 | 18 |
| Fatigue | 67 | 16 | 17 |
| Jaundice | 65 | 13 | 22 |
| Loss of appetite | 62 | 15 | 22 |
| Abdominal pain | 61 | 15 | 25 |
| Swelling in abdomen and legs | 61 | 13 | 26 |
| Easy bruising or bleeding | 42 | 26 | 32 |
| Tea-coloured urine | 27 | 29 | 44 |

**Table PS2** Impact of HCC in patients’ daily lives

| **Impact of disease** | **Respondents, %** | | |
| --- | --- | --- | --- |
|  | **All** | **Male** | **Female** |
| Significant | 39 | 36 | 48 |
| Moderate | 34 | 35 | 30 |
| Minimal | 10 | 12 | 5 |
| None | 17 | 17 | 18 |

**2.2.2 Patients’ experiences during the diagnosis of HCC**

**Table PS3** Time from first suspicion to diagnosis of HCC

| **Time taken, months** | **Respondents, %** | | |
| --- | --- | --- | --- |
|  | **All** | **Male** | **Female** |
| <1 | 26 | 26 | 25 |
| 1–3 | 42 | 44 | 40 |
| 4–6 | 13 | 10 | 20 |
| 7–12 | 6 | 4 | 10 |
| >12 | 12 | 16 | 5 |

**Table PS4** Communication with patient’s doctor or practice during the period of diagnosis

| **Communication** | **Respondents, %** | | |
| --- | --- | --- | --- |
|  | **All** | **Male** | **Female** |
| Excellent | 42 | 42 | 40 |
| Good | 44 | 43 | 48 |
| Neither good nor bad | 13 | 15 | 10 |
| Poor | 1 | 0 | 3 |
| Absent | 1 | 1 | 0 |

**Table PS5** Patients’ belief that they had received the correct medical and laboratory tests during the period of diagnosis

| **Patients thought they had received the correct tests** | **Respondents, %** | | |
| --- | --- | --- | --- |
|  | **All** | **Male** | **Female** |
| Yes | 85 | 82 | 93 |
| No | 3 | 2 | 5 |
| Don’t know | 12 | 16 | 3 |

**Table PS6** Person who first suspected HCC

| **Person** | **Respondents, %** | | |
| --- | --- | --- | --- |
|  | **All** | **Male** | **Female** |
| Hepatologist or gastroenterologist | 45 | 46 | 45 |
| General practitioner | 29 | 27 | 33 |
| Family member or friend | 7 | 10 | 0 |
| Oncologist | 7 | 3 | 15 |
| Other | 7 | 8 | 5 |
| Radiologist | 3 | 3 | 3 |
| Surgeon | 2 | 2 | 0 |

**Table PS7** Location of diagnosis

| **Location** | **Respondents, %** | | |
| --- | --- | --- | --- |
|  | **All** | **Male** | **Female** |
| Public hospital | 72 | 71 | 78 |
| Private hospital | 26 | 27 | 23 |
| Other | 2 | 2 | 0 |

**Table PS8** Contact with patient association or support group

| **Contacted a patient association or support group** | **Respondents, %** | | |
| --- | --- | --- | --- |
|  | **All** | **Male** | **Female** |
| Yes | 6 | 4 | 10 |
| No | 82 | 88 | 70 |
| Don’t know | 12 | 8 | 20 |

**Table PS9**  Clarity of information received by patients during period of diagnosis

| **Information was clear** | **Respondents, %** | | |
| --- | --- | --- | --- |
|  | **All** | **Male** | **Female** |
| Yes | 81 | 84 | 75 |
| No | 12 | 10 | 13 |
| Don’t know | 8 | 6 | 13 |

**Table PS10** Patients’ knowledge of first treatment received for HCC

| **Patient knows first treatment** | **Respondents, %** | | |
| --- | --- | --- | --- |
|  | **All** | **Male** | **Female** |
| Yes | 67 | 70 | 63 |
| No | 4 | 3 | 3 |
| Don’t know | 29 | 27 | 35 |

**Table PS11** Number of treatments (excluding surgery) received by patient

| **Treatments received, n** | **Respondents, %** | | |
| --- | --- | --- | --- |
|  | **All** | **Male** | **Female** |
| 0 | 9 | 8 | 13 |
| 1 | 44 | 44 | 45 |
| 2 | 34 | 33 | 35 |
| 3 | 9 | 11 | 5 |
| ≥4 | 4 | 4 | 3 |

**Table PS12** Communication with patient’s doctor or practice during the treatment period

| **Communication** | **Respondents, %** | | |
| --- | --- | --- | --- |
|  | **All** | **Male** | **Female** |
| Excellent | 37 | 42 | 28 |
| Good | 46 | 43 | 53 |
| Neither good nor bad | 17 | 16 | 20 |
| Poor | 0 | 0 | 0 |
| Absent | 0 | 0 | 0 |

**Table PS13** Patients’ treatment was stopped

| **Treatment was stopped** | **Respondents, %** | | |
| --- | --- | --- | --- |
|  | **All** | **Male** | **Female** |
| Yes | 16 | 13 | 20 |
| No | 84 | 87 | 80 |

**Table PS14**  Reason for stopping treatment

| **Reason** | **Respondents, %** | | |
| --- | --- | --- | --- |
|  | **All** | **Male** | **Female** |
| Treatment not affordable | 48 | 33 | 63 |
| Side-effects | 43 | 33 | 63 |
| Other specified | 19 | 25 | 13 |
| Lack of efficacy | 14 | 8 | 13 |
| No treatment available | 14 | 8 | 25 |
| Other (specify) | 14 | 17 | 13 |
| Clinical trial available | 5 | 8 | 0 |
| New treatment available | 5 | 0 | 13 |

**Table PS15**  Party responsible for the decision to stop treatment

| **Party responsible** | **Respondents, %** | | |
| --- | --- | --- | --- |
|  | **All** | **Male** | **Female** |
| Patient | 52 | 42 | 63 |
| Doctor | 43 | 58 | 25 |
| Other | 5 | 0 | 13 |

**Table PS16** Treatment outcome most important to patients

| **Treatment outcome** | **Respondents, %** | | |
| --- | --- | --- | --- |
|  | **All** | **Male** | **Female** |
| Cure | 53 | 57 | 43 |
| Extend life expectancy | 26 | 22 | 35 |
| Improve QoL | 14 | 13 | 15 |
| Avoid troublesome side-effects | 6 | 6 | 8 |
| Other | 1 | 1 | 0 |

**Table PS17** Agent providing support to patient

| **Agent** | **Respondents, %** | | |
| --- | --- | --- | --- |
|  | **All** | **Male** | **Female** |
| Doctor | 62 | 58 | 70 |
| Nurse | 35 | 26 | 55 |
| Family member or carer | 33 | 27 | 45 |
| Patient association or support group | 11 | 8 | 15 |
| Other | 7 | 9 | 3 |

**Table PS18** Patients experiencing treatment side-effects

| **Patient experienced side-effects** | **Respondents, %** | | |
| --- | --- | --- | --- |
|  | **All** | **Male** | **Female** |
| Yes | 56 | 52 | 63 |
| No | 44 | 48 | 37 |

**Table PS19** Patients’ pre-treatment awareness of treatment side-effects

| **Patient aware of side-effects** | **Respondents, %** | | |
| --- | --- | --- | --- |
|  | **All** | **Male** | **Female** |
| Yes | 68 | 71 | 65 |
| No | 32 | 29 | 35 |

**Table PS20**  Patients’ perspective of factors needing improvement during treatment

| **Factor to improve** | **Respondents, %** | | |
| --- | --- | --- | --- |
|  | **All** | **Male** | **Female** |
| Access to the most suitable treatment | 35 | 28 | 48 |
| Management of side-effects | 30 | 27 | 35 |
| Communication by doctor | 22 | 24 | 20 |
| Access to imaging | 18 | 13 | 28 |
| Availability of patient association or group support | 15 | 13 | 20 |
| Other^a^ | 14 | 13 | 15 |

^a^ Other factors specified by patients:

- Patient to understand: explain the cause and how to treat it
- Information about treatment outside Asia
- Distance from the hospital
- Connection with other patients
- Faster validation of the diagnosis
- Reduce patient discomfort

**Table PS21**  Patients’ health insurance coverage

| **Patient has insurance** | **Respondents, %** | | |
| --- | --- | --- | --- |
|  | **All** | **Male** | **Female** |
| Yes | 61 | 62 | 60 |
| No | 39 | 38 | 40 |

**Table PS22**  Affordability of HCC treatments to patients

| **Treatment is affordable** | **Respondents, %** | | |
| --- | --- | --- | --- |
|  | **All** | **Male** | **Female** |
| Yes | 47 | 52 | 38 |
| No | 53 | 48 | 63 |

**Table PS23**  Patients’ belief that they have access to the best possible treatment

| **Best treatment is accessible** | **Respondents, %** | | |
| --- | --- | --- | --- |
|  | **All** | **Male** | **Female** |
| Yes | 76 | 75 | 78 |
| No | 4 | 3 | 5 |
| Don’t know | 20 | 21 | 18 |
